# Sialic acid-modified phosphorene for low-necrosis and targeted drug delivery in breast cancer

**DOI:** 10.1039/d6ra03857c

**Published:** 2026-07-09

**Authors:** A. Souri, N. Khazamipour, O. Babaee, B. Dadashnia, M. Shojaie, A. Zandi, A. Mahjoori, M. Habibi-Rezaei, S. Mohajerzadeh

**Affiliations:** a Thin Film and Nanoelectronic Lab, School of Electrical and Computer Engineering, University of Tehran Tehran Iran mohajer@ut.ac.ir; b Protein Biotechnology Research Lab (PBRL) School of Biology, College of Science, University of Tehran Iran

## Abstract

Targeted drug delivery continues to face significant challenges in cancer therapy, necessitating innovative materials and methods that enhance treatment efficacy while minimizing systemic toxicity. In this study, we introduce a straightforward and solvent-free method for synthesizing black phosphorus (BP) nanosheets directly on silicon substrates *via* hydrogen-plasma treatment, followed by immediate functionalization with sialic acid (SA) molecules. This technique yields a stable, biocompatible phosphorene-based platform (called DSAP), characterized by its high loading capacity for the anticancer drug doxorubicin (DOX). The functionalization and stability of the nanosheets were rigorously evaluated using Raman spectroscopy, electron microscopy, X-ray photoelectron spectroscopy (XPS), atomic force microscopy (AFM), and high-resolution transmission electron microscopy (HR-TEM). The functionalized nanosheets demonstrated enhanced stability against degradation in aqueous environments. Notably, DSAP exploits Siglec–SA interactions for receptor-mediated uptake, ensuring precise tumor targeting. Zeta potential measurements confirmed efficient DOX adsorption onto DSAP. *In vitro* studies using MDA-MB-231 breast cancer cells revealed a significant enhancement in apoptotic cell death upon treatment with DSAP compared to treatment with mere DOX, as evidenced by high early and late apoptosis rates and substantially reduced necrosis. Electron microscopy provided a visual confirmation of these apoptotic effects, capturing distinct cellular features, such as membrane blebbing and chromatin condensation, indicative of receptor-driven programmed cell death. By uniting the innate biodegradability of BP nanosheets with the specificity imparted by SA, DSAP offers a transformative leap forward in cancer nanomedicine, potentially extendable to additional therapeutic approaches (*e.g.*, photothermal or photodynamic therapies) without sacrificing biocompatibility. These results position DSAP as a next-generation platform for precision drug delivery, paving the way for safer, more effective, and highly personalized cancer treatments.

## Abstract 2

Sialic-acid-functionalized phosphorene nanosheets enable targeted, pH-responsive doxorubicin delivery and induce apoptosis in MDA-MB-231 breast cancer cells.

## Graphical abstract

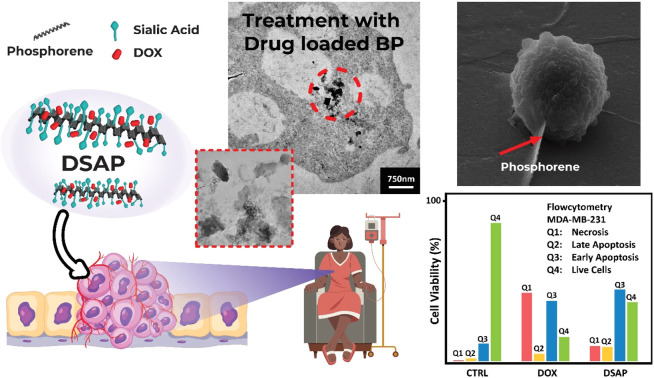

## Introduction

To date, an increasing interest in the novel medical applications of 2D nanomaterials with extraordinary physicochemical properties has led to a proliferation of research activities in the development of 2D nanocarriers for multimodal nanomedicine.^1–3^ For instance, graphene,^4^ Bi_2_Se_3_ (ref. 5), WS_2_ (ref. 6), and MoS_2_ (ref. 7) have been utilized to construct combination therapy platforms, showing their outstanding performance in targeted drug delivery. Two-dimensional nanomaterials have become even more attractive as biodegradability associated with the newly emerged members adds to their favorable properties.^8^ Apart from its biocompatibility, the unique degradability of phosphorene in physiological environments makes it an attractive 2D material for clinical applications, such as photodynamic therapy, photothermal therapy,^9,10^ drug delivery,^11,12^ biosensing^13,14^ and theranostics.^15,16^ Compared with other 2D materials, such as graphene and MoS_2_, BP has a much higher surface-to-volume ratio due to its puckered lattice configuration, which can increase the drug loading capacity. On the other hand, due to its unique electronic structure, BP is found to be a highly efficient photosensitizer and can be applied as a photodynamic therapy (PDT) agent to generate singlet oxygen.^17^ In addition, both BP nanoparticles and BP quantum dots show broad optical absorptions across the entire visible light region, making them suitable candidates for near-infrared (NIR) photo-thermal therapy (PTT).^3,18^

Despite the unique and favorable properties of black phosphorous for biological applications, BP faces serious challenges that limit its practical use. One major concern is the chemical exfoliation process involving toxic solvents like *N*-methyl-2-pyrrolidone (NMP).^19,20^ Moreover, the process of isolating and purifying BP from these chemical solutions is both labor-intensive and inefficient, adding complexity to its preparation. Another significant problem is the inherent instability of BP, which originates from its tendency to degrade when exposed to ambient conditions, such as air and moisture. This degradation can lead to changes in its chemical structure, reducing its effectiveness in biological systems. Therefore, finding stable, non-toxic methods for BP exfoliation and developing strategies to prevent its degradation are essential to enhance its viability for biological and medical applications.^19–22^

Cancer is a leading global health challenge, and effective treatment depends not only on early detection but also on targeted drug delivery strategies. Cancer cells exhibit unique glycan structures that differ from normal cells, playing crucial roles in processes such as cell proliferation, invasion, and metastasis.^23,24^ These glycan alterations, especially the overexpression of SA^25,26^ or hyper-sialylation, enable cancer cells to escape from the immune system and to promote tumor growth. Siglec receptors, a subset of lectins expressed on cancer cells, play a crucial role due to their ability to bind SA.^27,28^ Members of the Siglec family have been explored as potential therapeutic targets in cancer treatment, as they can facilitate the delivery of cytotoxic agents specifically to malignant cells;^29,30^ they have been studied as therapeutic targets in cancer for many years.^31^ The specific interaction between Siglecs and SA provides opportunities for precision drug delivery, enhancing the effectiveness of therapeutic interventions while minimizing off-target effects.^32–34^

Understanding the glycan–lectin interactions is essential for developing advanced, targeted drug-delivery technologies aimed at improving cancer treatment outcomes. To achieve targeted delivery, engineered nanocarriers or drug-loaded sheets leverage the high affinity of Siglecs for SA to bind selectively to cancer cell surfaces. Once attached, cellular entry typically occurs *via* receptor-mediated endocytosis,^35^ wherein the Siglec–SA interaction facilitates the internalization of the carrier–drug complex. This process allows the therapeutic agents to bypass non-targeted pathways, increasing drug accumulation within cancer cells while minimizing systemic side effects. Moreover, the acidic tumor microenvironment often enhances the release of therapeutic agents from nanocarriers post-internalization, further improving the efficacy of the treatment.^36,37^

A conventional well-established anticancer drug called doxorubicin or DOX, for short, has been used to induce cell death in cancer cells.^38,39^ Targeted delivery systems play a crucial role in directing DOX specifically to tumor cells, aiming to reduce its detrimental impact on healthy tissues and enhance therapeutic effectiveness. Based on our previous studies,^40–42^ we select phosphorene as a carrier for our drug delivery system. SA is chosen as a targeting moiety enhancer due to its significant ability to bind to hyper-sialylated ligands on cancer cells. An especially exciting aspect of our approach lies in the convergence of black phosphorus's tunable biodegradability and the sophisticated, receptor-mediated targeting enabled by SA. By uniting these two powerful concepts, we anticipate a transformative leap forward in precise cancer management.

In contrast to conventional drug-delivery methods that rely on passive accumulation and the risk of off-target toxicity, our DSAP platform directly capitalizes on Siglec–SA interactions to achieve highly specific binding, reducing collateral damage to healthy tissues. Not only does this cutting-edge strategy offer a broad therapeutic window for potent chemotherapeutics, like doxorubicin, but it also paves the way for future multimodal regimens, including photothermal or photodynamic therapies, without compromising biocompatibility. We envision that these unique attributes of DSAP, including simplicity of synthesis, high loading capacity, and robust chemical stability, hold immense promise for scaling into clinical solutions poised to redefine personalized cancer treatment. In this paper, we introduce a targeted drug-delivery system (DDS) called DSAP, which aims to enhance the effectiveness of cancer treatment in two ways: (1) using SA to target cancer cells specifically and (2) using BP as a non-toxic and biodegradable carrier for DDS. The main goal of our current work is to assess the effectiveness of DSAP as a DDS and to investigate the ability of SA to enhance the penetration of DOX-BP conjugates for intracellular delivery.

## Experimental section

### Material synthesis

In this study, we present a one-step, cost-effective, and reproducible method for the growth and crystallization of BP nanosheets on silicon substrates, eliminating the need for exfoliation from bulk material. Utilizing a novel direct-current plasma-enhanced chemical vapor deposition system (DC-PECVD), featuring two temperature zones and two sets of electrodes, we precisely controlled the deposition parameters and crystal formation. The dual-electrode setup uniquely enabled the simultaneous deposition of red phosphorus and immediate conversion of the amorphous film into highly crystalline, two-dimensional phosphorene structures. Specifically, the incorporation of hydrogen plasma facilitated the transport of phosphorus atoms from the source material onto the silicon substrates, while energetic hydrogen ions generated in the plasma enhanced adatom rearrangement, improving the crystallinity and uniformity of the resulting nanosheets.

The resulting high-quality 2D sheets are suitable for biological and electronic applications and can be readily passivated with various biomolecules. Although demonstrated on silicon, this method is adaptable to other substrates, such as glass and mica. Immediately after crystallization, the silicon substrates coated with BP sheets were immersed in a SA solution, initiating chemical interactions between SA molecules and phosphorus atoms. The functionalized SA-BP system presented three notable advantages. First, the freshly synthesized nanosheets offered abundant surface sites for efficient biomolecular binding. Second, this approach avoided toxic solvents, enhancing compatibility for biological uses. Lastly, the presence of SA maintained nanosheet stability under acidic conditions, effectively promoting pH-sensitive drug release mechanisms advantageous for targeted tumor therapy. Electrostatic interactions between BP and DOX further facilitated controlled drug delivery, specifically within acidic tumor microenvironments. Additional details regarding this single-step growth process are provided in the SI (Fig. S1).

**Fig. 1:**
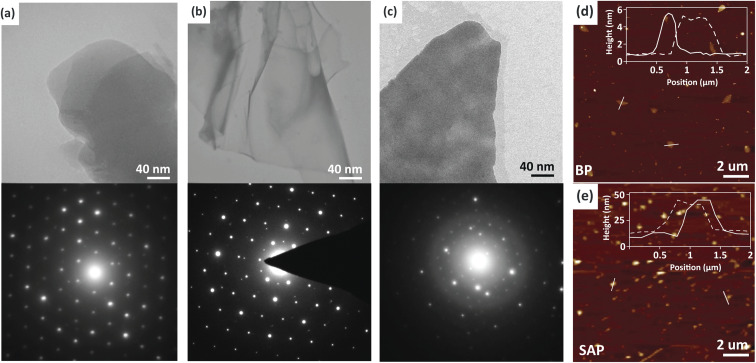
Characterization of the BP and SAP nanosheets before drug loading. TEM images and the corresponding selected area electron diffraction (SAED) patterns of (a) pristine BP nanosheets, (b) SAP nanosheets after sialic acid functionalization, showing the intact morphology with slightly modified diffraction patterns due to surface modification, and (c) DOX-loaded SAP nanosheets with visible morphological changes, with the dark regions suggesting drug accumulation. AFM images and the corresponding height profiles of (d) pristine BP nanosheets with an average height of ∼5 nm, indicating a few-layer structure and (e) SAP nanosheets after functionalization, showing an increased thickness (∼50 nm) and reduced lateral dimensions due to sonication during functionalization.

Fig. 1 shows the results of various material investigations on pristine, functionalized and loaded sheets. In part (a), one can have a general glance at the bright-field image of the puckered arrangement of phosphorene sheets. Fig. 1b depicts the bright-field image of SA-treated phosphorene sheets. The images in part “c” (Fig. 1c) highlight the loading of the sheets with doxorubicin. The presence of dark regions on the sheet surfaces in this part corresponds to the molecular attachment of doxorubicin to the phosphorene features. The electron diffraction pattern images across all regions confirmed that phosphorene retained its crystalline structure. In addition, the layered arrangement of these sheets was examined using AFM, where the formation of few-layered structures was corroborated. The AFM images showed the evolution of ultra-thin phosphorene nanosheets, with an average lateral size of 500 nm and a thickness of 5 nm (Fig. 1d). Subsequently, through passivation with SA, the SAP sheets exhibited an augmented thickness and reduced lateral size attributed to the sonication step in the ultrasonic process, resulting in average dimensions of 200 nm and 50 nm (Fig. 1e).

**Fig. 2:**
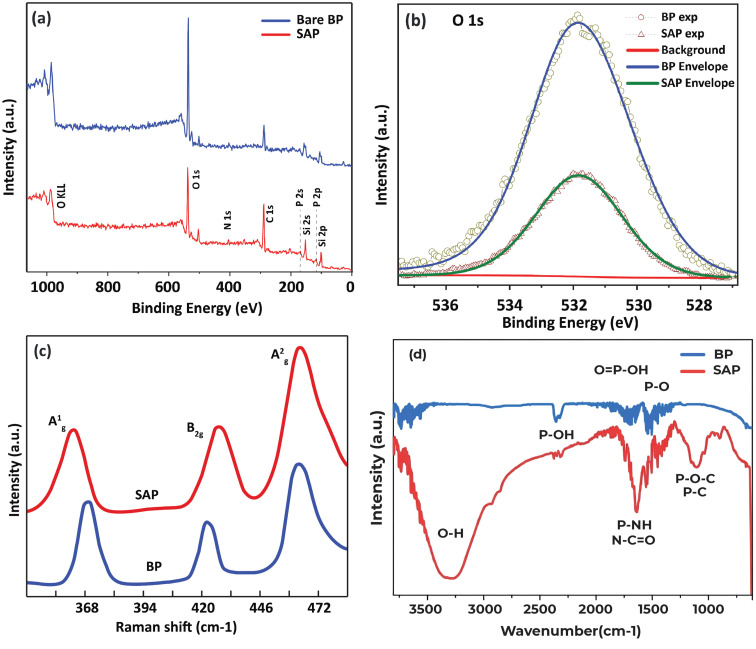
Chemical composition and bond formation during the functionalization of phosphorene with sialic acid (SAP). (a) Deconvolution of phosphorus species, showing a shift of the P 2p_1/2_ photoemission peaks towards higher binding energies, reflecting the greater oxidation of pristine BP *versus* the functionalized sheets. (b) High-resolution XPS spectra of the O 1s region, showing an oxygen-related peak at 532 eV. The SA-functionalized sheets tend to remain intact upon exposure to water or air, demonstrating efficient passivation by SA molecules. (c) Raman spectra of the BP and SAP sheets, displaying the three characteristic phonon modes (A^1^_g_, B__2g__, and A^2^_g_). The SAP spectrum exhibits red shifts, especially in the B__2g__ mode, attributed to the lattice strain induced by SA functionalization. (d) FTIR spectra of SAP and BP sheets, providing evidence for the formation of chemical bonds between phosphorene and sialic acid. The lower spectrum (SAP) shows a pronounced peak associated with P–O–C and P–C bonds, whereas this feature is absent in the upper BP spectrum.

Fig. 2 presents recent findings on the chemical composition of phosphorene sheets, followed by activation with SA. X-ray photoelectron spectroscopy (XPS), a robust analytical method,^37^ was utilized to examine BP flakes before and after SA treatment. The results, depicted in part (a), highlight a prominent peak at approximately 531.82 eV, corresponding to oxygen species. The high-resolution spectra provided in Fig. 2b further illustrate the photoemission peaks for O 1s at this binding energy, indicative of the oxygen functional groups of SA and the partial oxidation of the SAP sheets. Additional detailed spectra are available in the SI. Quantitative XPS analysis was conducted to compare the surface composition of the BP and SAP sheets by measuring the intensity of photoelectrons at precise binding energies. The results revealed that BP contained approximately 42.5 atomic percent (at%) oxygen, while the SAP sample exhibited a reduced oxygen content of about 24.98 at%. This substantial decrease confirmed the higher oxidation level in BP than in SAP. The chemical binding of SA molecules to available phosphorus atoms in the sheets reduced their interaction with oxygen molecules in the ambient environment, thereby lowering the surface oxygen concentration in SAP sheets. These XPS findings corroborated the effective passivation of phosphorene sheets by SA molecules, which stabilized the surface and prevented degradation even after prolonged exposure to air or aqueous solutions. This stabilization underscored the utility of SA-functionalized phosphorene in biological and chemical applications requiring enhanced material durability.

In part (c) of this figure, we have provided the Raman spectroscopy results pertaining to the phosphorene sheets, prior to and after passivation with SA biomolecules. The main peaks attributed to A^1^_g_, B_2g_ and A^2^_g_ phonons were located at the wavenumbers of 364, 429 and 465 cm^−1^ (SAP RAMAN shift), respectively. The A^1^_g_ peak corresponded to out-of-plane vibrations, whereas the other two peaks were related to in-plane phonons. As observed in the Raman spectra of both samples, the functionalization of the phosphorene sheets with SA led to a noticeable shift in the spectra compared with that of bare phosphorene features. While the A^1^_g_ and A^2^_g_ modes did not change significantly, the B_2g_ mode exhibited a marked alteration, from 422 to 429 cm^−1^. This shift was believed to be induced by the presence of large SA molecules attached to the sheets. The strong peaks at these high wavenumbers suggested that the passivated sheets exhibited enhanced stability relative to bare phosphorene.

To further confirm the successful functionalization of the phosphorene nanosheets, Fourier transform infrared (FTIR) spectroscopy was performed (Fig. 2d). The FTIR spectrum of SAP exhibited clear differences compared with that of bare BP, providing additional evidence of surface modification. In particular, the appearance of a broad absorption band in the 3200–3500 cm^−1^ region was attributed to O–H stretching vibrations from the hydroxyl and carboxyl groups of SA, while peaks near ∼2900 cm^−1^ corresponded to C–H vibrations. Moreover, characteristic bands observed around ∼1200 cm^−1^ were assigned to P–O–C and P–C bonds, indicating the formation of chemical linkages between SA molecules and the phosphorene surface. These features were absent or significantly weak in pristine BP, confirming successful functionalization (C21).

In addition to experimental characterization, theoretical simulations were conducted to further validate the stability and interaction mechanism between SA and phosphorene. The calculated adsorption energy (*E*_ads ≈ −0.37 eV) confirmed favorable and stable binding, while charge density difference analysis and Bader charge calculations (≈0.84 |e| transfer) revealed significant electronic coupling between SA molecules and the phosphorene surface. Furthermore, projected density of states (PDOS) analysis demonstrated clear orbital hybridization, supporting the formation of strong interfacial interactions. These combined experimental and theoretical results provided a comprehensive molecular-level understanding of the DSAP system, confirming its chemical stability, resistance to degradation, and suitability as a robust nanoplatform for drug-delivery applications (Fig. S5) (C13).

**Fig. 3:**
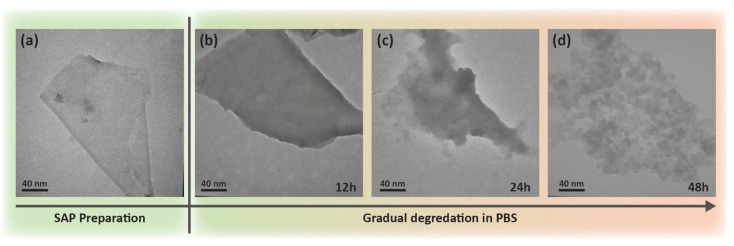
Stability and biodegradation behavior of the SA-functionalized phosphorene (SAP) nanosheets. (a) TEM image of the SAP nanosheets in an SA solution after one week, showing intact and well-defined sheet-like morphology, indicative of a stable and passivated structure prior to biological exposure. Time-resolved TEM images of the SAP nanosheets dispersed in PBS, mimicking physiological conditions, after 12 h (b), 24 h (c), and 48 h (d). The nanosheets retain their structural integrity at early time points (12 h), followed by progressive fragmentation and morphological degradation at 24–48 h. This controlled degradation demonstrates that SA functionalization provides sufficient short-term stability for cellular uptake while enabling subsequent biodegradation, a key feature for efficient intracellular drug release.

To directly evaluate the stability and degradation behavior of the SAP nanosheets under physiological conditions, over-time TEM analysis was performed in phosphate-buffered saline (PBS) (Fig. 3). As shown, the SAP nanosheets retained their structural integrity during the initial period (up to 12 h), maintaining a well-defined sheet-like morphology suitable for cellular interaction and uptake. Upon prolonged exposure (24–48 h), gradual morphological degradation was observed, characterized by fragmentation and the loss of structural continuity. This behavior indicated that SA functionalization provided sufficient short-term stabilization to preserve nanosheet integrity prior to cellular internalization while enabling controlled biodegradation over time. Such a balance between initial stability and subsequent degradation is critical for drug delivery applications, ensuring the effective transport of the therapeutic payload, followed by its release within the target cellular environment (C15).

### Drug loading

As mentioned before, bare phosphorene has a large surface area, making it an excellent nanocarrier for drug loading and delivery. Rather than primarily relying on π–π stacking (as graphene derivatives do), BP's lone-pair-mediated binding, surface/edge reactivity, and layered structure make it a strong candidate for drug loading and delivery. It offers multiple interaction pathways (hydrogen bonds, electrostatic attraction, and covalent functionalization), along with a good surface area, all of which support stable association with therapeutic molecules. As a result, the functionalized SAP can be considered as a promising carrier for DOX loading, a suitable choice for targeted drug delivery, since it bears unique features, such as non-toxicity and suitability for drug release. In this work, we systematically investigated the loading and release dynamics of BP-drug nanocomposites. Notably, SAP exhibited a negative charge in aqueous environments.

**Fig. 4:**
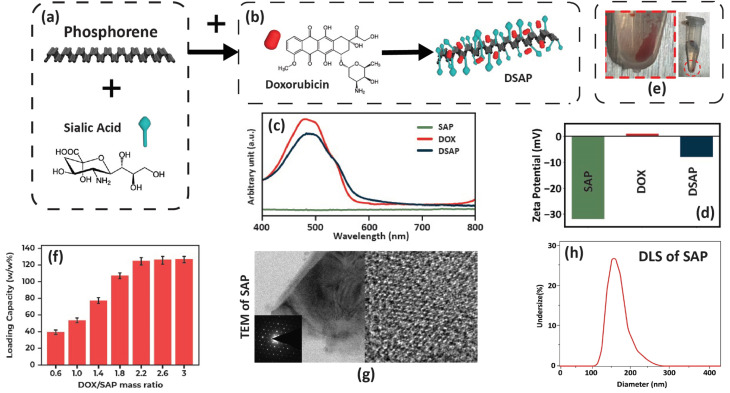
Schematic and experimental validation of the BP nanosheet functionalization and DOX loading. (a) Illustration of the functionalization process, with SA molecules being conjugated to the phosphorene sheets to form SAP, enhancing the biocompatibility and targeting ability. (b) Molecular structure of DOX and a model of its interaction with the phosphorene surface. Photographic evidence of red coloration in DSAP dispersions supports the visible drug loading. (c) UV-vis absorption spectra of SAP, DOX, and drug-loaded SAP (DSAP). (d) Zeta potential analysis showing the SAP nanosheets possessing a highly negative surface charge becoming less negative upon DOX loading, confirming the successful electrostatic interactions between SAP and the positively charged DOX molecules. (e) Photographs of the drug-loaded SAP nanosheets (DSAP), showing the characteristic red color after doxorubicin loading. (f) Loading capacity of SAP. (g) HRTEM images of the SAP nanosheets, confirming the preserved layered morphology and crystalline structure after sialic acid functionalization. Inset: SAED pattern, further verifying the structural integrity of the phosphorene nanosheets. (h) Dynamic light scattering (DLS) size distribution of the SAP nanosheets in an aqueous dispersion, showing a dominant hydrodynamic diameter in the range of approximately 150–200 nm, confirming their nanoscale dispersion and suitability for drug delivery applications.

The release behavior of DOX from BP nanosheets was investigated in other works^18,43–46^ under acidic conditions at a pH value of 5.0, reflecting the tumor microenvironments (TMEs), where the acidic nature of cancerous cells enhanced drug release. Small drug molecules with a positive charge possess the potential to be encapsulated within these interlayer spaces through electrostatic interactions. Importantly, while previous studies have demonstrated the pH-dependent release behavior of DOX from phosphorene-based systems under acidic conditions, the DSAP platform is fundamentally designed to exploit this well-established mechanism rather than to function under physiological pH conditions. We speculate that the strong electrostatic interactions between negatively charged SAP sheets and positively charged DOX molecules ensure high loading stability under neutral physiological environments, thereby minimizing premature drug leakage during systemic circulation. Upon exposure to acidic conditions, such as those encountered in tumor microenvironments and intracellular endo-/lyso-somal compartments, protonation effects weaken these interactions, triggering efficient drug release. While our investigation has been focused on an acidic environment (pH of 5), the drug release under neutral conditions (specifically pH values of 5.0 and 7.4) is an important parameter, and this behavior needs to be further investigated. The DSAP system is believed to fulfill the criteria of a stimulus-responsive nanocarrier, with functional validation evidenced through its therapeutic performance rather than comparative release profiles alone (C11). As a result, we selected DOX, a frequently administered drug for chemotherapy in clinical applications, as a model drug to be loaded onto the BP nanosheets.^47–49^Fig. 4a provides the atomic arrangement of the phosphorene sheets prior to and after attachment to the SA molecules in a schematic fashion. After loading the functionalized sheets with DOX, they were used to treat malignant breast cells.

Furthermore, zeta potential analysis revealed a notable change in the surface potential of the SAP nanosheets, shifting from −30 mV to −8.5 mV after loading with DOX, as illustrated in Fig. 4d. This shift in the average zeta potential to −8.5 mV could be attributed to the positive charge of DOX, which partially neutralized the negative surface charge of the SAP nanosheets. This was believed to be an indication of the appropriate and efficient loading of the drug on the surface of functionalized sheets. The presented photos in part (e) correspond to the sample after being loaded with the desired drugs. The highly loaded sample was well observed with large flakes of nanosheets in a red color. UV-vis spectroscopy was employed to assess drug loading, as depicted in Fig. 4c. The loaded DOX exhibited a similar absorption peak as the free DOX, indicating interaction with BP nanosheets and confirming successful DOX loading. All these results indicated the successful loading of DOX onto the SAP surface.

Drug loading was quantitatively evaluated using drug loading content (DLC) and drug loading efficiency (DLE, also referred to as encapsulation efficiency, EE). DLC was defined as the mass of DOX loaded relative to the SAP carrier mass 
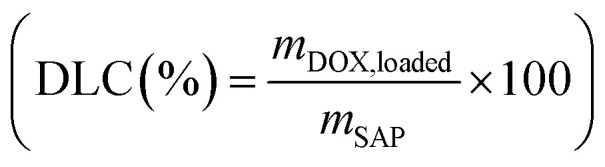
, while DLE represents the fraction of the initially added DOX that was successfully loaded 
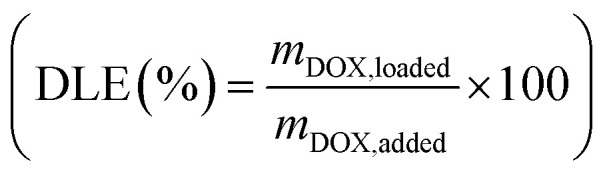
. Using a DOX/SAP feed mass ratio of 1 : 8 with SAP at 150 µg mL^−1^, the total DOX added was 270 µg mL^−1^. The amount of non-loaded (free) DOX in the supernatant was determined using UV-vis spectroscopy. A calibration curve was first constructed from standard DOX solutions at different concentrations, yielding a linear relationship between absorbance and concentration, which was then used to quantify the residual DOX. The loaded DOX was subsequently calculated by subtracting the free DOX from the initial amount added. Based on the experimentally determined loading capacity at this ratio (107% w/w relative to SAP), the amount of loaded DOX was 160.5 µg mL^−1^, corresponding to a DLC of 107% and a DLE of 59.4%. This mass ratio (1.8) was selected as the optimal loading condition (Fig. 4f), as it provided a high drug payload while maintaining a reasonable loading efficiency, consistent with previously reported high-surface-area nanocarrier systems, where elevated drug-to-carrier ratios could be achieved without substantial loss in loading performance (C14). To further investigate the interaction of sialic acid with the phosphorene sheets, we examined the functionalized nanostructures using high-resolution transmission electron microscopy, where the lattice arrangement of the phosphorene sheets was evident. As an interesting observation in this figure, one can see the presence of sialic acid molecules extended on the surface of the phosphorene sheets (Fig. 4g). Finally, in part (h), we have provided the DLS data of the SAP solutions, indicating a range of nanosheets between 150 and 200 nm.

## Results: biocompatibility and therapeutic response

**Fig. 5:**
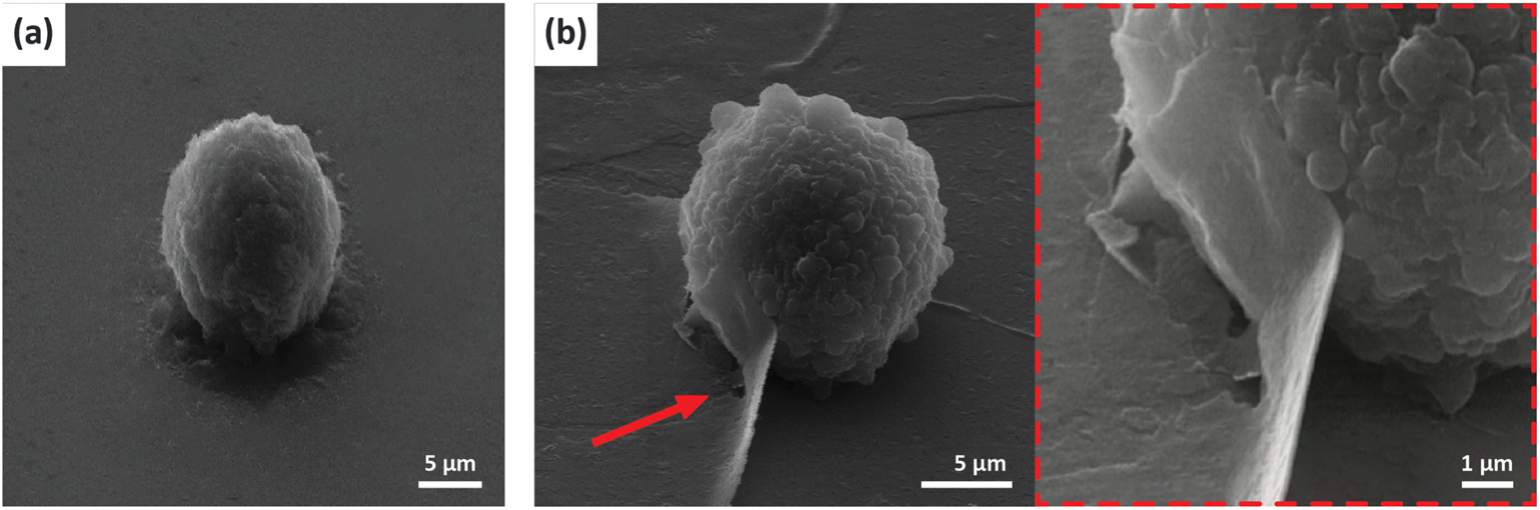
SEM images showing the adhesion behavior of a cancer cell on different surfaces. (a) MDA-MB-231 cells cultured on a bare surface, exhibiting minimal interaction with the substrate. (b) MDA-MB-231 cells on a phosphorene-modified surface, demonstrating strong attachment to the sheet. The red arrow shows a part of the cell extending toward the phosphorene sheet, indicating a strong interaction. The inset (red-dashed box) provides a high magnification view, revealing detailed cell adhesion structures.

First, the biocompatibility of BP nanosheets was assessed using an MTT cytotoxicity assay. As illustrated in Fig. S2, the results revealed viability above 95%. Additionally, the BP nanosheets exhibited no significant cytotoxicity toward MDA-MB-231 cells, even at concentrations as high as 150 µg mL^−1^. Given the exceptional characteristics of DSAP, further investigations were conducted to evaluate its cellular uptake mechanisms and *in vitro* therapeutic effects. To investigate the interaction between cell membranes and SAP, we developed a novel experimental approach. As depicted in Fig. 5a and b, SAP was applied to the surface to facilitate the capture of cancer cells, based on our hypothesis that this process was mediated by the interaction between SA residues and Siglec-15 receptors present on the cancer cell membrane. For this study, two identical surfaces were prepared, one coated with SAP and the other left uncoated. Fig. 5a highlights the lack of interaction on the uncoated surface, serving as a control.

Cancer cells were cultured on both surfaces, and SEM imaging (Fig. 5b) confirmed the attachment of cancer cells to the SAP-coated surface, suggesting a strong affinity between the cells and the nanomaterial. These findings highlighted the potential of SAP-coated surfaces in biosensor applications for cancer cell detection. Based on our previously submitted paper (A novel high-sensitivity electrochemical sensor for cancer-cell detection by means of phosphorene sheets functionalized by sialic acid bio-molecules), Siglec-15 overexpression has been widely reported in invasive tumor cells compared to noninvasive normal cells,^27^ contributing to enhanced adhesion, migration, and invasion *in vitro*. To further validate the specificity of the Siglec–SA interaction as the underlying targeting mechanism, additional control experiments were considered and incorporated into the analysis (refer to our recent work^42^). In particular, a non-targeted control system (BP-DOX without SA functionalization) was evaluated, which demonstrated significantly reduced interaction efficiency with cancer cells, confirming the essential role of SA in mediating selective binding. Moreover, comparative studies were performed using different cell models with varying levels of invasiveness and receptor expression. In addition to MDA-MB-231 cells, which are known to overexpress Siglec-15 receptors, MCF-7 cells were employed as a less aggressive breast cancer model with comparatively low receptor expression. The observed reduction in targeting efficiency and cellular response in MCF-7 cells further supported the receptor-mediated nature of the interaction. Complementary evidence was obtained from electrochemical impedance spectroscopy (EIS) measurements (Fig. S6) using a SAP-modified working electrode, where a significantly higher charge transfer resistance (*R*_ct_ ≈ 3.25 kΩ) was observed for MDA-MB-231 cells compared to non-cancerous HUVEC cells (≈520 Ω), indicating stronger binding affinity due to the Siglec–SA interactions. In contrast, bare electrodes exhibited markedly low sensitivity and selectivity. Collectively, these results provided converging evidence from both biological assays and electrochemical analysis, demonstrating that the DSAP system achieved selective targeting through Siglec-mediated recognition, thereby validating its mechanism of action despite the inherent experimental limitations in accessing additional non-tumorigenic cell lines (C12).

**Fig. 6:**
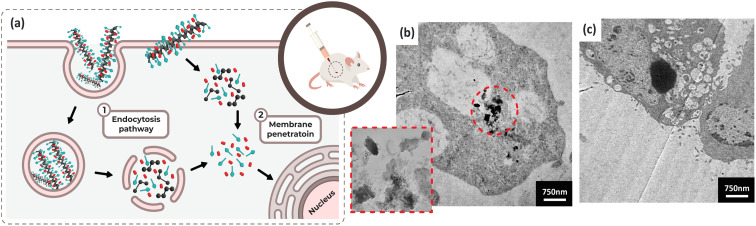
Cellular uptake and intracellular behavior of the DOX-loaded SA-functionalized phosphorene (DSAP) nanosheets. (a) Schematic of two proposed mechanisms for cellular entry: (1) receptor-mediated endocytosis and (2) direct membrane penetration. After internalization, the nanosheets release the drug into the cytoplasm, allowing it to reach the nucleus and inhibit cancer cell proliferation. (b) Transmission electron microscopy image of the MDA-MB-231 cell. The dark features in these images pertain to the imported phosphorene nanosheets, loaded with DOX. (c) TEM image of a cancer cell 24 hours after DSAP treatment, displaying multiple indicators of apoptosis. Observable features include cytoplasmic shrinkage, membrane blebbing, chromatin condensation near the nucleus, and vesicular structures associated with apoptotic body formation, all consistent with regulated, programmed cell death induced by the DSAP system.

The interaction of loaded sheets with cancerous cells is depicted in Fig. 6a; BP-based delivery platforms have been extensively reported to enter cells predominantly *via* endocytic pathways, involving mechanisms such as clathrin-mediated endocytosis, caveolae-mediated endocytosis, and macropinocytosis. These pathways ensure the encapsulation of the BP nanosheets within vesicles, such as endosomes, following cellular uptake. However, emerging evidence suggests that the BP nanosheets, owing to their unique structural and physicochemical properties, may also facilitate direct membrane penetration by interacting with the lipid bilayer.^18,43,50,51^ We proposed that DSAP was delivered into the cytoplasm through both direct membrane penetration and endocytosis, followed by endosomal escape as the ultra-thin BP nanosheets underwent biodegradation.

Fig. 6(b and c) demonstrates that a treated sample was prepared for cross-sectional TEM imaging, revealing nanosheets' penetration into cells, as shown in part (b) of this figure. Part (c) shows the following 24 hours of DOX treatment; based on the TEM cross-sectional images, several apoptotic indicators are observed. These included cell shrinkage, a hallmark of apoptosis, where the cytoplasm became condensed and less voluminous. Additionally, membrane blebbing was evident, with parts of the cell membrane bulging outward, further indicating the apoptotic process. Chromatin condensation was also visible, characterized by tightly packed genetic material appearing as dark regions near the nucleus. Lastly, the presence of vesicular bodies suggested the formation of apoptotic bodies, a classic feature of cells breaking down in preparation for removal by immune cells.^52,53^

To further evaluate the cytotoxicity profile and concentration-dependent behavior of the developed nanoplatform, standard MTT assays were performed for both unloaded and drug-loaded systems. As shown in Fig. S8(a), the control, SA, and SAP groups maintained high cell viability (>95%), confirming the negligible intrinsic toxicity of both SA and the SAP nanocarrier. In contrast, free DOX exhibited a pronounced cytotoxic effect, reducing cell viability to approximately 16%, while the DSAP system demonstrated a moderated yet effective cytotoxic response, indicating controlled drug delivery. Importantly, this distinction highlighted that the observed cytotoxicity in DSAP arose primarily from DOX activity rather than carrier-induced effects.

**Fig. 7:**
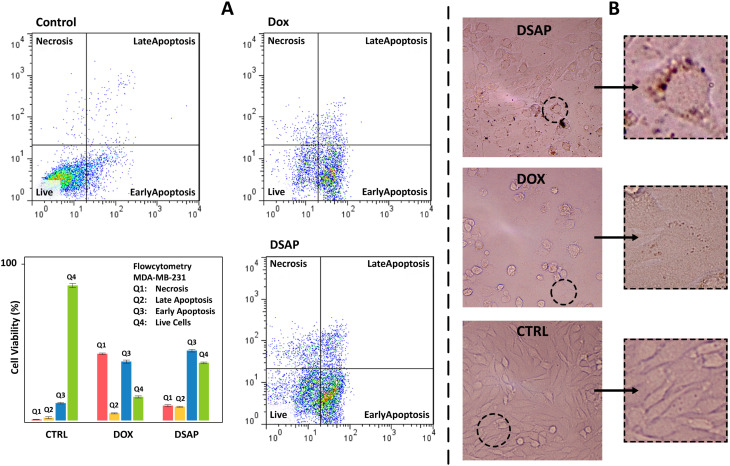
(A) Results of flow cytometry on three groups of assays: control, DOX and DSAP mixtures. The number of cells has shown a severe drop in the case of DOX, indicating a high level of lysed cells for this drug system. By counting the number of cells for all three experiments, the total necrosis level for the DOX system is around 42%, which is much higher than that for the DSAP system. It is deduced that the DSAP system leads to early and late apoptosis, which further corroborates the effectiveness of the targeted drug delivery. (B) Optical images revealing the necrotic morphology in DOX-treated cells *versus* the controlled apoptotic features in DSAP-treated cells.

Furthermore, the concentration-dependent cytotoxicity of SAP was systematically evaluated, as presented in Fig. S8(b). Even at increasing concentrations (100, 125, and 150 µg mL^−1^), the SAP nanosheets exhibited minimal toxicity, with cell viability consistently remaining above ∼95%. This confirmed the excellent biocompatibility of the unloaded nanocarrier across a relevant concentration range. When considered alongside the flow cytometry results (Fig. 7A), which demonstrated enhanced apoptosis in the DSAP-treated cells, these findings collectively verified that the SAP platform was inherently safe, while the DOX-loaded system provided effective and controlled therapeutic activities. Such behavior fulfills a key requirement for advanced drug delivery systems, where the high biocompatibility of the carrier is coupled with efficient, concentration-dependent drug action (C23).

As shown in Fig. 7A, three experimental groups have been prepared for flow cytometry analysis as control, DOX, and DSAP. Each group was seeded with 7000 cells, counted using a hemocytometer. After 24 hours of incubation, the cells were treated with either DOX or DSAP, both containing the same concentration of doxorubicin. Following an additional 24 hours of treatment, the samples were analyzed using flow cytometry, revealing notable differences in cell viability and the mechanisms of cell death among the groups. In the DOX-treated group, the total cell count was 40% lower than that of the control group (however, both had the same number of cells seeded at first), indicating significant cell lysis, which was not reflected in the necrosis region (Q1) of a flow cytometry chart. However, if these lysed cells were included in the necrosis rate, it would amount to 42.7%, demonstrating the severely destructive cytotoxic effects of DOX. In contrast, the DSAP-treated group showed a significantly low necrosis rate of 9.55%, underscoring the effectiveness of the targeted delivery system in reducing necrosis while maintaining therapeutic efficacy by leading the cells to an apoptotic state. Apoptosis is a regulated form of cell death that prevents inflammation and collateral tissue damage, making it a more favorable mechanism for cancer therapy compared to necrosis, which can trigger an inflammatory response, promote tumor progression, and reduce treatment efficacy.^54,55^

The late apoptosis (Q2) part of the flow-cytometry chart was elevated in both treatment groups compared to the control group (1.8%), with the DOX group showing 4.6% and the DSAP group exhibiting 8.8%. The high proportion of late apoptosis in the DSAP group suggested improved targeting and delivery, effectively inducing programmed cell death. Early apoptosis (Q3) also increased significantly in both the DOX (37.7%) and DSAP (44.7%) groups compared to the control (11.1%). Again, the high rate of early apoptosis in the DSAP group indicated enhanced drug uptake by cancer cells and the efficient activation of the apoptotic pathway. The proportion of live cells (Q4) was highest in the control group (86.4%), reflecting normal cell conditions. The DOX-treated group, however, showed a dramatic reduction in live cells to 15.1%, consistent with the strong cytotoxicity of the drug. In contrast, the DSAP group preserved more viable cells, with 36.9% remaining live, demonstrating reduced off-target effects and relatively high selectivity.

In conclusion, the DSAP system demonstrated superior targeted drug delivery compared to DOX alone. Flow cytometry data suggested that DSAP enhanced both early and late apoptosis, reduced necrosis, and resulted in more selective cancer cell death with fewer off-target effects. The high percentage of lysed cells in the DOX group underscored its non-specific toxicity, while the DSAP system provided a more controlled and selective therapeutic approach.

Fig. 7B provides optic microscopy images to illustrate the comparative morphology of cancer cells under three experimental conditions: untreated control, treated with DOX, and treated with DSAP. In the control group, the cancer cells exhibited typical spindle-shaped morphology with smooth and intact plasma membranes, indicative of healthy and adherent growth characteristics. In the DOX-treated group, cells appeared rounded, swollen, and detached, clearly displaying features of necrotic cell death, including noticeable disruption and perforation (holes) in the plasma membrane, highlighting compromised membrane integrity and uncontrolled cytoplasmic leakage. Conversely, cells in the DSAP-treated group displayed characteristics consistent with apoptotic cell death, evidenced by cell shrinkage, membrane blebbing, and the presence of distinct apoptotic bodies (cell death packages), reflecting a highly regulated and orderly cellular fragmentation process. The morphological evidence confirmed that while DOX treatment induced necrosis with pronounced membrane disruption and cellular swelling, DSAP treatment promoted apoptosis, leading to controlled fragmentation into apoptotic bodies, indicating a preferable, less inflammatory therapeutic action.

Although the present study provides a proof-of-concept validation of the DSAP nanosystem through synthesis, physicochemical characterization, and *in vitro* biological evaluation, further *in vivo* investigation is required to fully assess its translational potential. In particular, breast cancer animal models will be necessary to evaluate tumor-targeted accumulation, biodistribution, biocompatibility, and stimuli-responsive drug release under biologically complex conditions. Therefore, detailed *in vivo* studies are planned as a continuation of this research line to further validate the therapeutic performance of the developed nanocarrier system.

In Table 1, we have collected some of the available data on the use of phosphorene, with or without a specific linker, for interaction with biological species such as cancer cells or genomes. As provided in the table, our SAP formalism does not need an extra passivation step, nor does it use linker molecules. In contrast, the use of SA as a biomolecule acted both as the surface passivation agent and the agent for interaction with the surface of the cells or drug loading. The presence of SA on the phosphorene sheets helped their interaction with the Siglec 15 receptors, promoting their attachment to the cancerous cells. At the same time, the SA-functionalized surface of phosphorene was believed to be a suitable medium to anchor large drug molecules, such as DOX. Furthermore, the direct growth of phosphorene on a silicon substrate and the subsequent exfoliation in an aqueous sialic acid solution eliminate the need for NMP alcohol. The latter material is believed to be toxic, and avoiding this step is a great progress towards phosphorene-based chemotherapy.

**Table 1:** Phosphorene-based biological systems

Study/system	Linker/entrapment	Toxicity	Degradability	Preparation method
SAP for breast cancer therapy (this work)	SA (direct conjugation and linker-free)	Low toxicity (viability > 95% and minimal necrosis)	Stabilized by SA and degrades physiologically	Direct crystallization *via* DC-PECVD (solvent-free)
BP-DOX hydrogel^43^	Physical entrapment in hydrogel	Low (biodegradable and injectable)	Hydrogel promotes breakdown *in situ*	Liquid-phase exfoliation and uses NMP
BP-PEG prodrug^30^	Chemical linker (ester or amide bond with DOX)	High cytotoxicity in cancer and potential off-target effects	Prodrug degrades intracellularly	Liquid-phase exfoliation and uses NMP
Self-degradable BP for breast cancer therapy^45^	Direct BP interaction with Cas13a/crRNA	Low toxicity and specific inhibition of Mcl-1	Self-degradable BP (responsive to tumor environment)	Liquid-phase exfoliation and uses NMP
BP nanosheets for genome editing^46^	CRISPR/Cas9 loading	Controlled cytotoxicity and genome editing purposes	Endosomal escape and degradation	Liquid-phase exfoliation and uses NMP
BP as a chemo-photothermal agent^44^	Electrostatic interaction and surface adsorption	Moderate toxicity, enhanced by photothermal effects	Degradation enhanced by photothermal activation	Liquid-phase exfoliation and uses NMP
BP nanosheet system for synergistic PTT/PDT/CTT^18^	Electrostatic interactions	Low systemic toxicity with local responsiveness	Responsive to pH and NIR triggers	Liquid-phase exfoliation and uses NMP

## Summary and conclusions

In this study, we developed a novel SA-functionalized phosphorene-based drug-delivery platform (DSAP) through a simple, solvent-free fabrication approach, enabling the efficient and targeted delivery of doxorubicin (DOX) to breast cancer cells. The integration of phosphorene's unique physicochemical properties with the biological specificity of SA provided a multifunctional nanoplatform capable of both high drug loading and receptor-mediated targeting *via* Siglec interactions. Comprehensive material characterization, including XPS analysis and theoretical simulations, confirmed the successful functionalization, enhanced surface stability, and strong molecular interactions between SA and phosphorene, addressing key limitations associated with the intrinsic instability of black phosphorus.

Biological evaluations demonstrated that the DSAP system significantly enhanced therapeutic efficacy compared to free DOX. Flow cytometry and microscopy analyses revealed increased early and late apoptosis, alongside a marked reduction in necrosis, indicating a shift toward controlled, programmed cell death. Importantly, cytotoxicity studies confirmed that the unloaded SAP nanocarrier exhibited negligible toxicity across a wide concentration range, while the DOX-loaded system displayed a concentration-dependent therapeutic effect. This distinction highlighted the inherent biocompatibility of the carrier and the effectiveness of the drug delivery mechanism. Furthermore, the targeting capability of the DSAP platform was validated through a combination of biological and electrochemical analyses. The presence of SA enabled selective interaction with Siglec receptors overexpressed on invasive cancer cells, resulting in enhanced cellular uptake and specificity. Comparative studies using different cell models, along with electrochemical impedance spectroscopy, demonstrated the ability of the SAP-modified system to distinguish between cancerous and non-cancerous cells, confirming the role of receptor-mediated targeting in improving selectivity.

When compared with existing phosphorene-based and conventional DOX delivery systems, the DSAP platform exhibited several key advantages. Unlike systems requiring complex synthesis routes, chemical linkers, or toxic solvents, such as *N*-methyl-2-pyrrolidone (NMP), DSAP was fabricated *via* a straightforward and environmentally friendly process. The SA functionalization not only enhanced targeting but also provided effective surface passivation, significantly improving stability against oxidation and degradation. In addition, DSAP demonstrated a favorable balance between high drug-loading capability, controlled pH-responsive release, low carrier toxicity, and enhanced therapeutic selectivity. These combined features distinguish DSAP from previously reported systems that often suffer from instability, off-target effects, or fabrication complexity.

Overall, the DSAP system represents a promising next-generation nanoplatform for targeted cancer therapy, offering a synergistic combination of stability, biocompatibility, and selective drug delivery. The design strategy presented here provides a scalable and versatile framework that can be extended to other therapeutic agents and multimodal treatment approaches, including photothermal and photodynamic therapies. These findings highlight the potential of SA-functionalized phosphorene as a powerful tool in advancing precision nanomedicine and improving clinical outcomes in cancer treatment (C22).

## Conflicts of interest

There are no conflicts to declare.

## Supplementary Material

RA-OLF-D6RA03857C-s001

## Data Availability

The data supporting this article have been included as part of the supplementary information (SI). Supplementary information: additional experimental details, supporting physicochemical characterization data, and *in vitro* results for the phosphorene, SAP, and DSAP nanosheets. See DOI: https://doi.org/10.1039/d6ra03857c.
