# Supplementary material for: Sialic acid-modified phosphorene for low-necrosis and targeted drug delivery in breast cancer
Source: RSC Adv. 2026 Jul 9. Online ahead of print. doi: 10.1039/d6ra03857c (PMC13346871; doi:10.1039/d6ra03857c)
Supplement: RA-OLF-D6RA03857C-s001 [file RA-OLF-D6RA03857C-s001.pdf]

Supplementary Information (SI) for RSC Advances.
This journal is © The Royal Society of Chemistry 2026

Supporting Information

Sialic Acid–Modified Phosphorene for Low-Necrosis, Targeted Drug Delivery in Breast Cancer

*1A. Souri, 1N. Khazamipour, 1O. Babaee, 1B. Dadashnia, 1M. Shojaie, 3 A. Zandi,1A. Mahjoori, 2 M.Habibi and 1S. Mohajerzadeh**

1- Thin Film and Nanoelectronic Lab, School of Electrical and Computer Engineering, University of Tehran, Tehran, Iran

2- Protein Biotechnology Research Lab (PBRL) School of Biology, College of Science, University of Tehran, Iran

* Corresponding author: mohajer@ut.ac.ir

EXPERIMENTAL SECTION

Materials. Red Phosphorus powder purchased from Merck ≥97%, Hohenbrunn, Germany. Sialic acid (N-acetylneuraminic acid) was obtained from Merck.

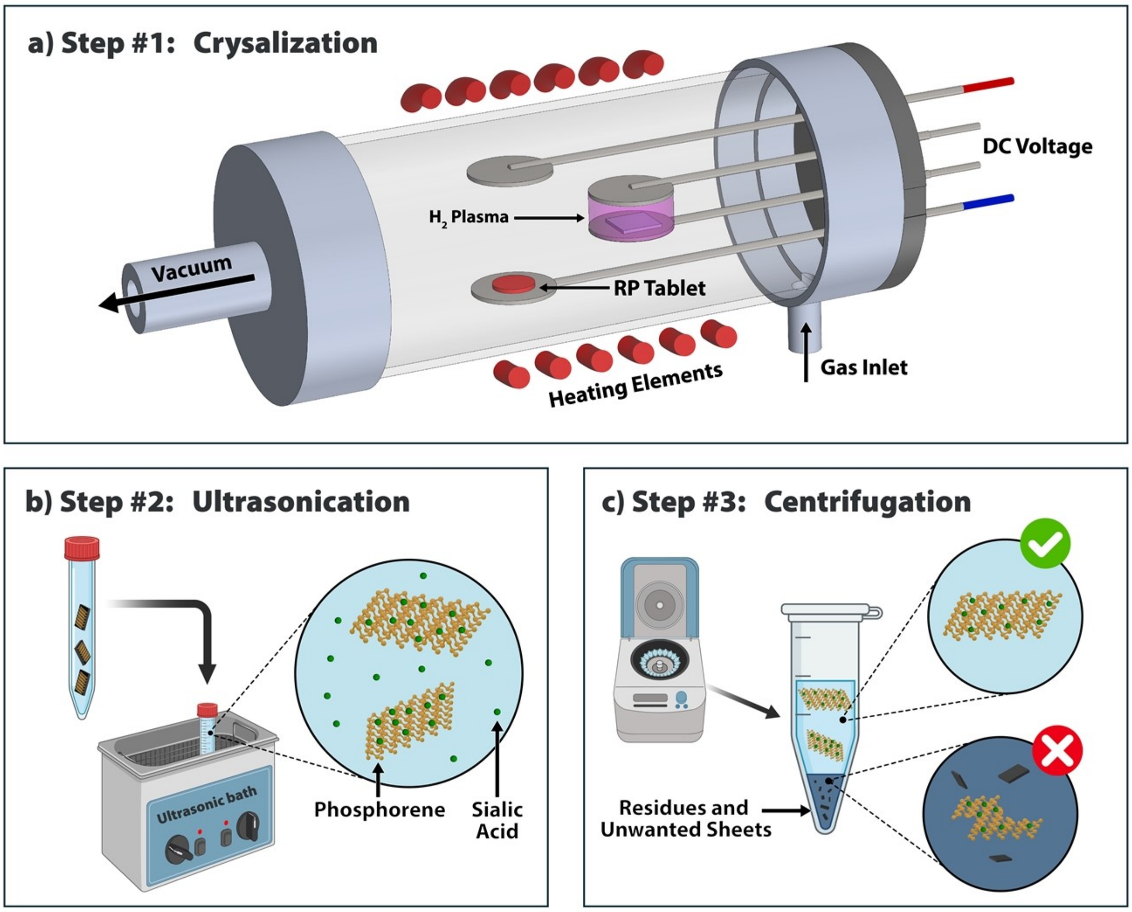

Figure S1: The general setup used for the realization of phosphorene nanosheets directly on silicon substrates followed by immersion in an aqueous solution, and further separation of desired functionalized sheets. (a) The growth reactor containing separate electrodes at hot (middle) and cold zones of the quartz cylinder. The gas inlet allows the inflow of desired gas combination while the vacuum system at the other end of the reactor establishes proper pressure. (b) immersion and agitation of the silicon substrates which are coated with phosphorene sheets in a sonication bath to peel off the sheets from the substrate and help for a chemical binding between the suspended biomolecules in the solution with fresh phosphorus atoms on the sheets. (c) Separation of the desired functionalized sheets using a centrifugation at 2000 rpm, where the top solution contains proper SAP sheets.

Synthesis and Preparation of SA-Functionalized BP Nanosheets. The formation of phosphorene sheets is based on our previously reported direct crystallization and thinning of red phosphorous layers and their conversion into phosphorene nanosheets[33]. To arrive at this stage, we exploit a direct-current plasma-enhanced chemical vapor deposition system (DC-PECVD) specifically designed for this purpose to adjust the growth temperature, various gases with controllable flows and highly adjustable plasma conditions. The fabrication commences by dicing Si wafers into small square pieces (1cm2) by means of a laser cutting unit, followed by cleaning in an RCA#1 solution, rinsing in D.I. water and blow drying with air.

The red-phosphorus (RP) tablet is made of 100 mg of phosphorus powder, pressed with a handy press. The prepared RP tablet is placed in the hot zone of the reactor (middle electrodes) to act as a source for phosphorus evaporation. The chamber is then evacuated to an initial pressure of 10mTorr, and the temperature rises to 370oC while a hydrogen gas flows to maintain the vacuum at 450mTorr. Once the desired temperature is reached, the main valve of the reactor is interchanged with a small bypass valve to rise the pressure and prepare the reactor for plasma ignition. Currently, the pressure of the reactor is around 2-3 Torr. During this period, the hydrogen gas acts as the carrier gas to help evaporation of RP and to form a thin and highly controllable layer of phosphorous on top of the silicon substrate(s). An internal DC power supply is used to apply voltage to the electrodes which hold the silicon substrates and to create a low energy plasma with an average of 1.5W/cm2 onto the silicon pieces. The plasma condition highly depends on temperature, vacuum level,

gas flow, and plasma power. Hydrogen gas with a flow rate of 10sccm entered the chamber when the vacuum reaches a base pressure of 10mTorr, and simultaneously the temperature was set to 370oC to apply plasma treatment for 2 minutes and off for 1 minute and repeat this sequence for 10 times. This temperature is set at a value to avoid re-evaporation of the previously deposited RP layer, while providing the required condition for an effective crystallization of randomly placed phosphorus atoms.

Sialic Acid (SA) has been exploited to functionalize the freshly prepared phosphorene layers on silicon substrates. For this purpose, we have used 0.3 mg/ml of SA in water to make a 1.5 ml solution. As previously stated (Fig. 1a), the formation of phosphorene sheets is feasible through conversion of amorphous film into highly crystalline sheets, directly on the silicon substrates[35]. Once the crystallization is complete, the silicon substrates which hold BP sheets on, are immediately immersed in the SA solution to initiate the interaction of sialic molecules with the phosphorous atoms of the sheets. To increase the concentration of functionalized sheets, we immerse several silicon pieces in 1.5 ml solution, just after the BP sheets have been formed in DC-PECVD reactor. In most cases, we have used four pieces of phosphorene-coated silicon samples to supply the phosphorene sheets within the sialic-containing solution. The solution which holds silicon wafers is placed in a bath sonication for 15 min at room temperature to exfoliate the phosphorene and suspend them within the solution. The exfoliated nanosheets are now exposed to sialic

molecules where the chemical binding between the fresh surfaces of the phosphorene and bio-molecules can be established. Following this step, the whole solution is poured in Falcon holders to be placed in a centrifugal apparatus. After proper centrifugation (2000 rpm, 10 min), the undesired residues of silicon substrates as well as improper thicker sheets will settle at the bottom of the holder. To avoid these undesired features, we pick up the top 1ml of the solution for future investigations.

Material characterization:

The physical properties of the phosphorene have been studied using a scanning electron microscope (Hitachi-S4160), operated at a voltage of 20kV. Moreover, the crystalline structure of the sheets has been deeply studied using Philips CM300 TEM. The surface morphology of the sheets has been further investigated using an atomic force microscope (NT-MDT AFM apparatus in noncontact mode with an NSG10 tip (190kHZ resonance frequency).

The optical behavior of the sheets has also been studied using a Teksan-Opus Raman spectroscope with a Nd: YAG green laser (λ=532 nm) at low power, ≈7mW, using a long work distance ×60(LWDx60) objective lens to avoid destructing the flakes. In addition, the electronic structures of elements were studied by X-ray photoelectron spectra (XPS, BESTEC) spectrometer using Al Kα (1486.6 eV) radiation as the source. Fourier transform infrared spectroscopy–attenuated total reflectance (FTIR-ATR) of the samples was obtained through Thermo Scientific (NICOLET iS10) spectrophotometer in the 4000–400 cm−1 range.

Ultraviolet-visible (UVvis) spectrophotometer (The PerkinElmer Lambda 25 UV/Vis Spectrophotometer) was used for UVvis absorbance measurements. Zeta-potential analyses were performed using zeta-potential analyzer (Perkin-Elmer lambda 25 spectrometer), and all data were averaged over three measurements.

Cell culture: The National Cell Bank of the Iranian Pasteur Institute is where the human breast cancer cell line MDA-MB-231 was purchased. These cells were cultured in Dulbecco's Modified Eagle Medium (DMEM, Sigma), 10% FBS, and 1% penicillin/streptomycin (Gibco) at 37 °C in a humidified incubator with 5% CO_2. PBS (pH 7.4), fetal bovine serum (FBS), DMEM, penicillin, and streptomycin were supplied by Aldrich.

MTT assay: First, the cytotoxicity was assessed using the MTT [3-(4,5-dimethylthiazol-2-yl)-5-diphenyltetrazolium bromide] assay (M2128, Sigma). Absorbance was assessed using a microplate reader at 570 nm (BioTek, UK).

Annexin V/PI viability assay: The Annexin V/PI viability assay was used to measure cell apoptosis and necrosis.

Toxicity analysis:

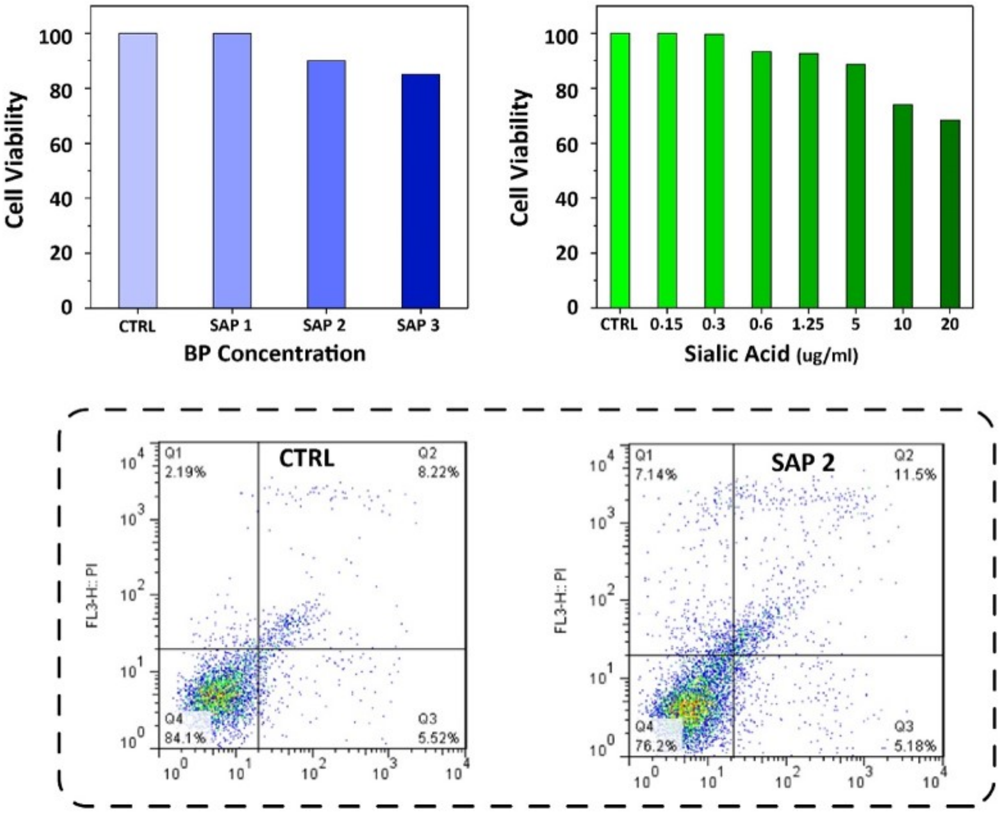

Figure S2. Toxicity assays: cytotoxicity assays of Sia-passivated sheets on MDA-MB-231cell line. (a) the results of MTT assays on cell viability for different concentrations of SAP. By raising the concentration, one can see a drop in viability to 72%. (b) Similar investigations with varying percentages of BP concentrations indicate a reduction in viability at higher values of BP. (c) flow cytometry data showing little toxicity once comparing the control data with the Sia-passivated sheet exposure[1].

Table S 1. Nanosheet concentration[1]

SAP1	SAP2	SAP3
50 ug/mL	150 ug/mL	300 ug/mL

To determine nanosheet concentration, exfoliated BPNShs in ethanol and dried them in a vacuum. Then BPNShs were weighted.

Cellular uptake:

Tumor cells can be distinguished from normal cells by unique features such as abnormal glycosylation, overexpression of specific receptors, and altered extracellular environments, which are crucial in oncogenesis, tumor progression, and metastasis. Recent advancements in glycomics have identified glycosylation as a therapeutic target, particularly through the binding of Siglec-15 on tumor cells to PB-SA. By functionalizing microelectrodes with BP-SA, our research group demonstrated effective cancer cell adhesion, confirmed by SEM imaging. Electrochemical studies showed that cancer cell attachment significantly increased the charge transfer resistance (Rct) of electrodes, with the Nyquist plot's semicircle expanding from 400 Ω to 3200 Ω, indicating the potential of BP-SA-based systems for cancer detection and targeted therapy.

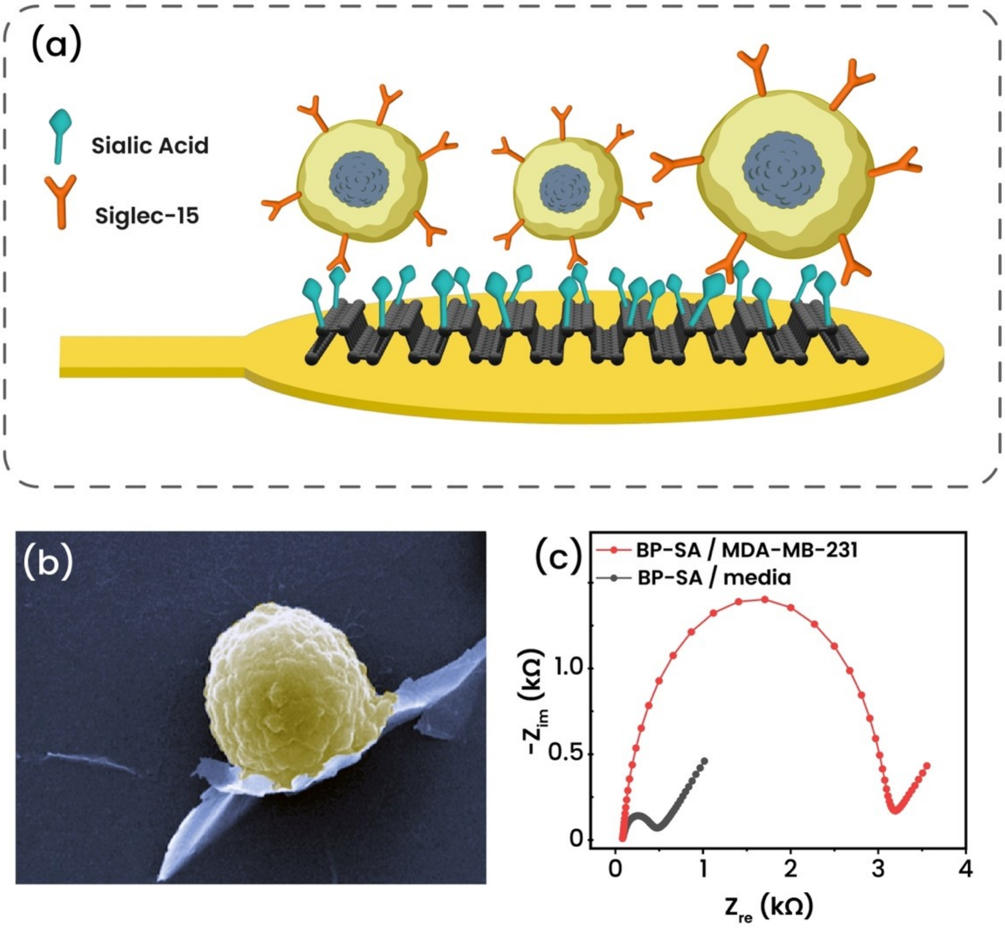

Figure S3. (a) Suggested schematic of cancer cell with the sialic biomolecules on the surface of phosphorene nanosheets. (b) A unique and unseen SEM image of a cancer cell attached on phosphorene sheets. The presence of SA molecules on the surface of phosphorene sheets would enhance cell settling on the sheet. (c) impedance spectroscopy response of two states of examination with and without cancer cells. An almost 10 times difference is observed in the result.

More details in Material Characterization for a Drug Delivery Platform

As shown in Fig. 4S, we performed XPS analysis to investigate the surface compositions and chemical states of the elements before and after functionalization of black phosphorus (BP) with sialic acid (SA). The XPS survey scans in Figure. 4S(a) revealed binding peaks for Si 2p, P 2p, O 1s, C 1s, and N 1s elements, with all data calibrated using the C 1s peak at 284.6 eV. The XPS spectra indicated a carbon peak at 284.7 eV, associated with the incorporation of sialic acid into the BP sheets, while the SAP sample showed an additional peak at 288.3 eV, attributed to carboxyl groups. The phosphorus peak at 133.45 eV was deconvoluted into two peaks corresponding to P 2p3/2 and P 2p1/2 states. Moreover, the O 1s peak at 532.2 eV confirmed the presence of oxygen functional groups from SA and partial oxidation of the SAP sheets.

Quantitative analysis of the XPS spectra revealed that the SAP sheets exhibited significantly lower oxygen content (21.4 at%) compared to BP (42.5 at%), confirming that the functionalization with sialic acid effectively reduced oxidation. The XPS data also showed a higher concentration of carbon in the SAP sample, attributed to the bio-molecules' carbon atoms. These results confirm the passivation of the phosphorene sheets with sialic acid, preventing degradation from prolonged exposure to air or aqueous environments, and are consistent with the expected formation of P-O-C or P-C bonds during the functionalization process. These findings provide strong support for the stability of the SAP system and corroborate our assertion that sialic acid functionalization enhances the surface stability of phosphorene, making it suitable for various applications, including drug delivery.

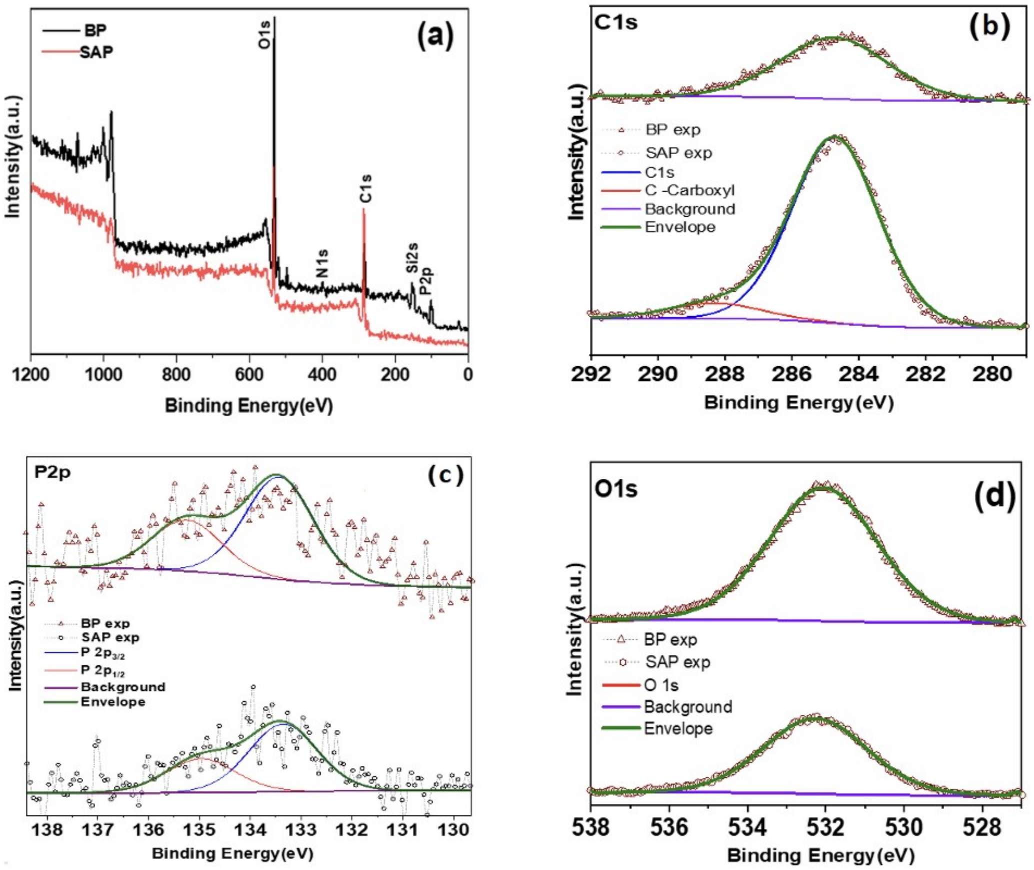

Figure S4: XPS analysis: Determination of surface composition and chemical state of different elements using X-ray photoelectron spectroscopy (XPS). (a) Wide-range survey spectrum indicating the presence of different elements, high-resolution spectra of (b)C1s, (c) P2p and (d) O1s[1].

In our previous work we have conducted a theoretical simulation to evaluate the stability and adsorption behavior of sialic acid (SA) molecules on phosphorene. The adsorption energy (Eads) was calculated to be -0.37 eV, indicating a stable binding between SA and phosphorene, categorized as covalent bonding. The adsorption configuration, shown in Fig. 5S(c), illustrates the optimal geometry where the carboxyl group of SA interacts with the phosphorene nanosheet at a distance of 2.53 Å, consistent with previous reports on similar systems. The charge density difference (Fig. 5S(d)) reveals significant electron transfer, with electron accumulation near the phosphorene surface and depletion near the SA molecule, suggesting strong coupling between the two. The Bader charge analysis confirms a charge transfer of 0.83746 |e|, indicating that the phosphorene surface becomes electron-enriched upon SA adsorption. Furthermore, the Projected Density of States (PDOS) in Fig. 5S(e) shows the hybridization and strong bonding between SA and phosphorene, with a HOMO-LUMO gap of 5.6 eV, which is relevant for potential applications such as sensors. The localized nature of the HOMO-LUMO levels (Fig. 5S (f)) on the carboxyl group of SA confirms its reactivity, especially in electron-donating processes. This analysis

demonstrates the stable adsorption of sialic acid on phosphorene and its potential impact on the electronic properties of the system, enhancing its suitability for various applications.

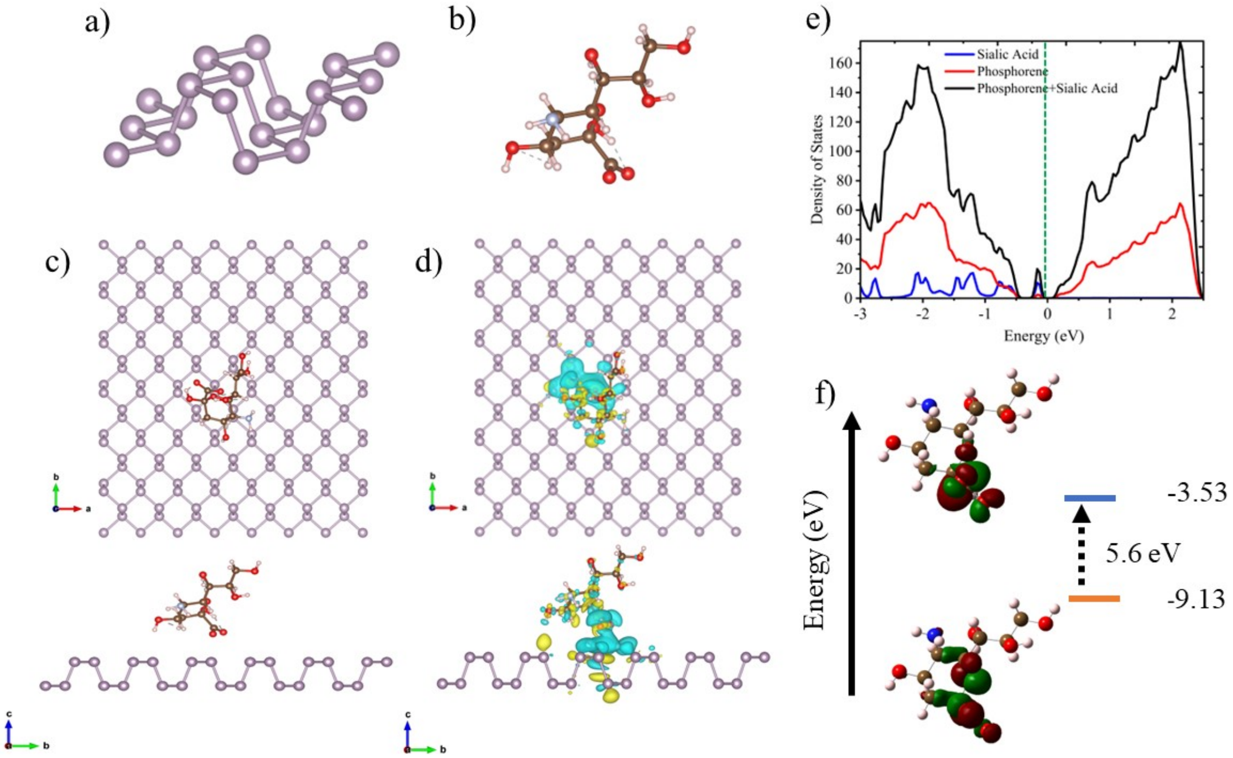

Figure 5S: (a) and (b) relaxed structures of phosphorene and sialic-acid molecule, respectively. c) top and side views of relaxed structure of SA molecule on top of phosphorene nanosheet. d) top and side views of spatial charge density of SA molecule on top of phosphorene nanosheet. e) projected density of states (PDOS) for SA molecule(blue), phosphorene nanosheet (red), and SA adsorbed on the phosphorene nanosheet (black). f) HOMO and LUMO energy levels for the SA molecule[2].

Electrochemical Test

we have fabricated an electrochemical sensor incorporating a sialic acid–modified working electrode (SAP-modified WE), which demonstrated significantly enhanced sensitivity compared to the bare working electrode. This improved performance is attributed to the functionalization of the electrode surface, enabling specific biological interactions. To evaluate the selectivity and sensing capability of the modified electrode, we conducted comparative electrochemical impedance spectroscopy (EIS) studies using MDA-MB-231 (highly invasive breast cancer cells) and HUVEC (non-cancerous endothelial cells) as a control. The ability of the modified WE to differentiate between these cell types was assessed by exposing them to the BP-SA-functionalized surface, followed by a 1-hour incubation period. As shown in Fig.S6 , the EIS response for MDA-MB-231 cells exhibited a significantly higher charge

transfer resistance (Rct ≈ 3.25 kΩ) compared to HUVEC cells (≈ 520 Ω). This marked difference is attributed to the overexpression of Siglec-15 receptors on MDA-MB-231 cells, which promotes stronger binding interactions with the SA moieties on BP-SA nanosheets. These findings clearly demonstrate the enhanced sensitivity and selectivity of the SAP-modified electrode over the bare electrode, particularly for invasive cancer cell detection.

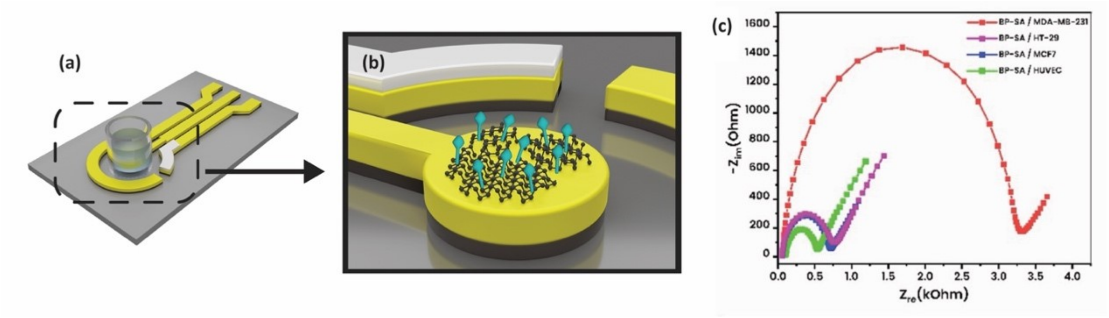

Figure S6. (a) Schematic illustration of the electrochemical sensing platform with a modified working electrode. (b) Magnified view of the electrode surface functionalized with BP–SA nanosheets, enabling selective interaction with target cells. (c) Nyquist plots obtained from electrochemical impedance spectroscopy (EIS) measurements, demonstrating enhanced charge transfer resistance for MDA-MB-231 cells compared to control cell lines (HUVEC and MCF7), indicating the sensor's high sensitivity and selectivity[2].

DLC and DLE

we have now included both Drug Loading Content (DLC) and Drug Loading Efficiency (DLE) to provide a comprehensive evaluation of the loading performance. These parameters were determined using UV–Vis spectroscopy by first constructing a calibration curve from standard DOX solutions at different concentrations to obtain a linear absorbance–concentration relationship. This calibration was essential for quantifying the amount of free (unloaded) DOX in the supernatant, from which the loaded DOX was calculated by mass balance. At a DOX/SAP mass ratio of 1.8 (SAP = 150 μg/mL), the loading capacity was determined to be 107% (w/w), corresponding to a DLC of 107% and a DLE of 59.4%. These results demonstrate that the SAP nanocarrier achieves a high drug payload while maintaining reasonable loading efficiency.

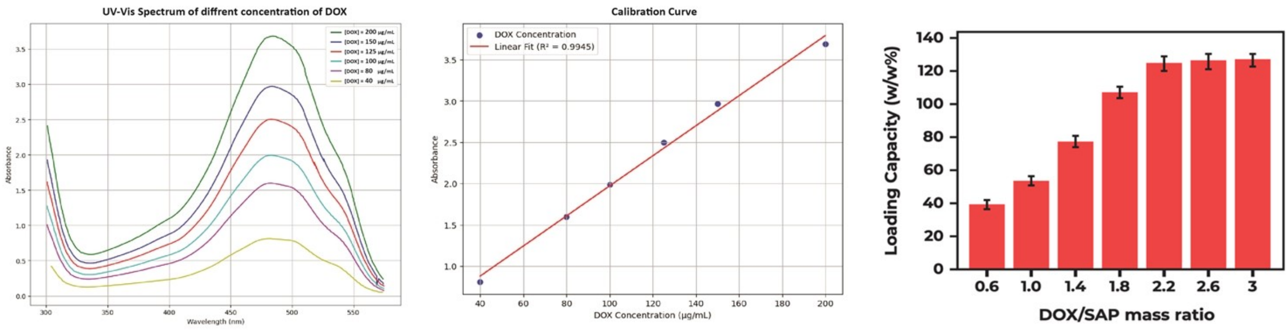

Figure S7. (Left) UV–Vis absorption spectra of DOX at different concentrations. (Middle) Corresponding calibration curve showing a linear relationship between absorbance and DOX concentration. (Right) Drug loading capacity of SAP nanosheets at varying DOX/SAP mass ratios, indicating saturation at higher ratios.

Cell Viability

As shown in Figure.S8, the cytotoxicity of SAP was assessed at various concentrations of BP (SAP 1, SAP 2, and SAP 3) and sialic acid (0.15 to 20 µg/mL). The results show that both SAP and SAP-loaded DOX systems exhibit low toxicity, with cell viability remaining above 85% even at the highest concentrations (SAP 3 and 20 µg/mL of SA).

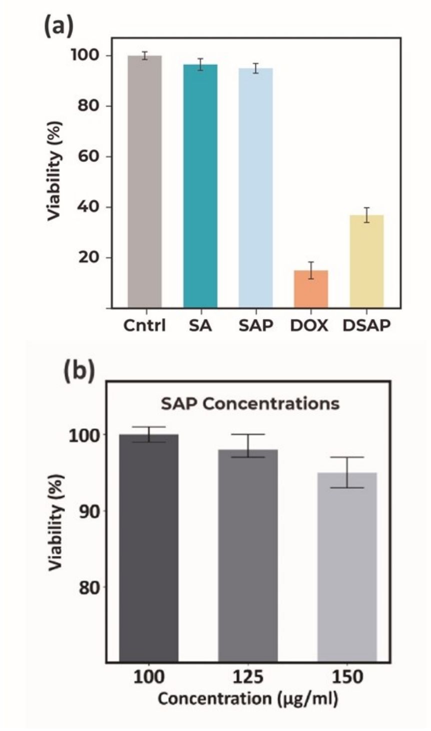

Figure S8. (a) MTT assay results showing cell viability after treatment with control, SA, SAP, free

DOX, and DSAP. The SA and SAP groups maintain high viability (>95%), confirming low intrinsic toxicity, while free DOX induces significant cytotoxicity. The DSAP system exhibits a moderated cytotoxic effect, indicating controlled drug release from the nanocarrier. (b) Cell viability following treatment with increasing concentrations of SAP (100–150 μg mL^{-1}), demonstrating minimal toxicity and confirming the excellent biocompatibility of the nanocarrier across the tested concentration range.

References:

1. Khazamipour, N., et al., *Linker-free Functionalization of Phosphorene Nanosheets by Sialic Acid Biomolecules.* Langmuir, 2024. **40**(13): p. 7067-7077.
2. Souri, A., et al., *A novel high-sensitivity electrochemical sensor for cancer-cell detection by means of phosphorene functionalized by Sialic-Acid Bio-molecules.* Nanoscale, 2025.
